# Rainbow trapping of ultrasonic guided waves in chirped phononic crystal plates

**DOI:** 10.1038/srep40004

**Published:** 2017-01-05

**Authors:** Zhenhua Tian, Lingyu Yu

**Affiliations:** 1 Department of Mechanical Engineering, University of South Carolina, Columbia, SC, 29208, USA

## Abstract

The rainbow trapping effect has been demonstrated in electromagnetic and acoustic waves. In this study, rainbow trapping of ultrasonic guided waves is achieved in chirped phononic crystal plates that spatially modulate the dispersion, group velocity, and stopband. The rainbow trapping is related to the progressively slowing group velocity, and the extremely low group velocity near the lower boundary of a stopband that gradually varies in chirped phononic crystal plates. As guided waves propagate along the phononic crystal plate, waves gradually slow down and finally stop forward propagating. The energy of guided waves is concentrated at the low velocity region near the stopband. Moreover, the guided wave energy of different frequencies is concentrated at different locations, which manifests as rainbow guided waves. We believe implementing the rainbow trapping will open new paradigms for guiding and focusing of guided waves. Moreover, the rainbow guided waves with energy concentration and spatial separation of frequencies may have potential applications in nondestructive evaluation, spatial wave filtering, energy harvesting, and acoustofluidics.

Rainbow trapping effect is originated from studies in electromagnetic waves that different frequency components can be slowed down and concentrated at different locations in the waveguide, thus allowing for temporary storages of lights, optical signal processing and enhanced light-matter interactions^1^,^2^,^3^,^4^,^5^,^6^,^7^,^8^,^9^,^10^. Inspired by the rainbow trapping of electromagnetic waves, research has also been conducted in trapping acoustic waves. Zhu *et al*. achieved rainbow trapping of acoustic waves through spatial modulation of sound velocity in tailored materials with perforated gradient grooves^11^. Ni *et al*. achieved it in a compact device made of space-coiling metamaterials^12^. Romero-García, *et al*. demonstrated sound rainbow trapping effect in chirped sonic crystals through spatial tuning of dispersion^13^. Also by adjusting dispersion in space, Jia *et al*. achieved the spatial separation of spoof surface acoustic waves on graded groove gratings^14^. Recently, Zhu *et al*. developed helical-structured acoustic metamaterials that can achieve sound deceleration without modulate the dispersion characteristics^15^. Despite the advances in controlling acoustic waves in air, there is few study about rainbow trapping of ultrasonic guided waves in solid waveguides to the best knowledge of the authors.

Ultrasonic guide waves are elastic waves propagating in different waveguides such as plate-like structures, pipes, structural surfaces, and interfaces^16^. Compared to traditional bulk waves, the guided waves have the advantage of long propagation distance with less energy loss^16^. With this merit, guided waves have been studied extensively for non-destructive evaluation^16^, structural health monitoring^17^, and acoustofluidics^18^,^19^,^20^,^21^. To achieve better performance in these applications, there is a growing interest in controlling the propagation of guided waves. Recently, research on waveguide design has shown great potentials to achieve innovative capabilities of guided waves, such as negative refraction, wave guiding, and wave focusing^22^,^23^,^24^,^25^,^26^,^27^,^28^,^29^,^30^,^31^,^32^. For example, Zhu *et al*. demonstrated negative refraction of S_0_ mode guided waves using single-phase metamaterials^25^. Wu *et al*. realized focusing of A_0_ mode guided waves through a gradient-index phononic crystal plate^26^. Yan *et al*. proposed surface bonded elastic metamaterials to focus A_0_ mode guided waves^28^. Philippe *et al*. presented a simple flat lens consisted of a thickness trough introduced into a plate, which can focus high frequency S_2_ mode through the mode conversion between the forward S_2_ mode and the backward S_2b_ mode at both edges of the trough^29^. Carrara *et al*. used arrays of aluminum stubs bonded on plates to focus guided waves for energy harvesting^32^. Although previous studies have demonstrated negative refracting, wave guiding and wave focusing, there is few study about rainbow trapping of guided waves except a recent simulation work that investigates broadband trapping of guide waves in plate using local resonances of metamaterials^33^.

Motivated by the chirped photonic and sonic crystals that have spatially variant dispersion properties and been used for rainbow trapping of electromagnetic^9^ and acoustic waves^13^, this study adopts one-dimensional (1D) chirped phononic crystals to achieve the rainbow trapping for ultrasonic guided waves. The numerical simulation and experiments demonstrate that the guided waves at different frequencies are slowed down and concentrated at different locations, which manifests as rainbow guided waves. Through dispersion analysis, it is found that the demonstrated rainbow trapping is related to the spatially variant stopband and the progressively slowing group velocity along the chirped phononic crystal plate.

## Results

**Figure 1:**
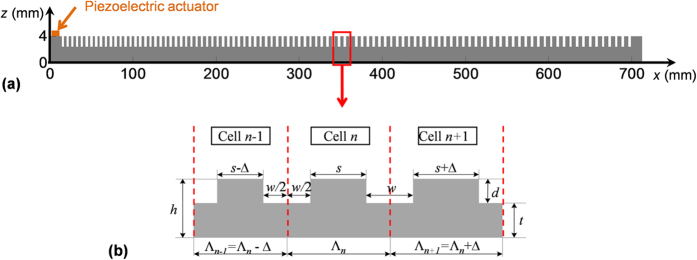
Schematics for a chirped phononic crystal plate. (**a**) The cross-section of the plate. (**b**) Three consecutive cells in the plate. All grooves in the plate have the same width *w* and depth *d*. The spacing *s* between two consecutive grooves is linearly increasing with the same increment *∆* along the +*x* direction.

In this study, we implement the rainbow trapping of ultrasonic guided waves using a chirped phononic crystal plate that spatially modulates the stopband and group velocity of the waves. The 1D chirped phononic crystal plate is made in a 4 mm thick aluminum plate with an array of 101 grooves. The cross-section of the plate is plotted in Fig. 1a, and three consecutive cells *n* − 1, *n* and *n* + 1 are plotted in Fig. 1b. All grooves have the same width *w* = 2.95 mm and depth *d* = 1.61 mm. Along the *x* direction of the plate, the cell length Λ_*n*_ linearly increases from 5.65 mm to 7.95 mm with the same increment *∆* = 0.023 mm.

### Dispersion analysis

In the chirped phononic crystals, the cell length linearly increases along the *x* direction. Since the increment *∆* between two consecutive cells is very small (0.023 mm), we could consider the *n*^th^ cell with length Λ_*n*_ in chirped phononic crystals as a cell with the same length Λ_*n*_ in uniform phononic crystals. Therefore, we could use dispersion characteristics of the uniform phononic crystals to approximate dispersion characteristics at the *n*^th^ cell in the chirped phononic crystals. For the uniform phononic crystals, the frequency-wavenumber dispersion relation *f(k*) can be derived by solving a modal analysis problem on a unit cell with Bloch-Floquet condition. Details of dispersion derivations and related analytical approximations can be found in the supplementary document.

**Figure 2:**
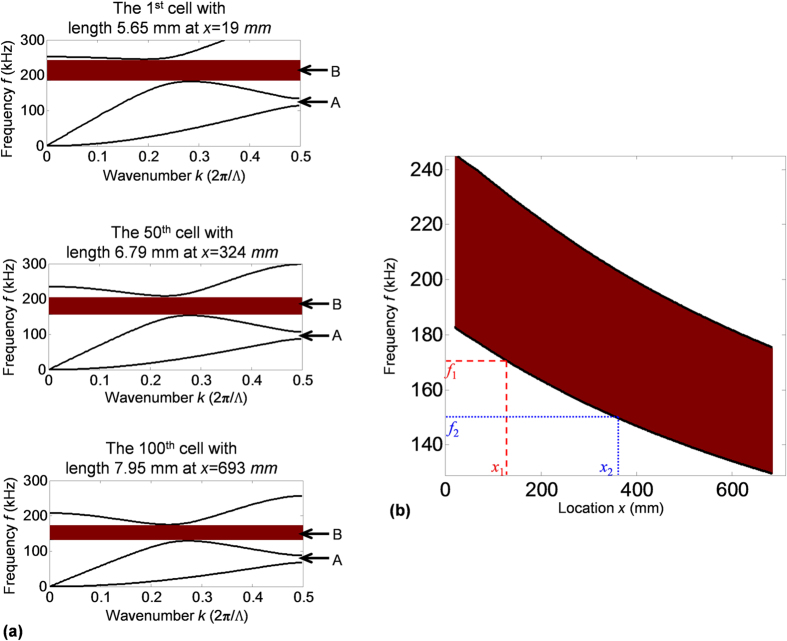
Dispersion analysis. (**a**) Local frequency-wavenumber dispersion curves in the first Brillouin zone for cells at the 1^st^ cell (top), 50^th^ cell (center), and 100^th^ cell (bottom) of the chirped phononic crystal plate. (**b**) Variation of local stopband with respect to location *x* in the chirped phononic crystal plate. The shaded areas represent stopbands.

Since the cell length Λ_*n*_ linearly increases along the *x* direction in the chirped phononic crystal plate, cells at different locations will have different frequency-wavenumber dispersion curves. To show the spatial evolution of dispersion curves in the chirped phononic crystal plate, Fig. 2a plots the frequency-wavenumber dispersion curves in the first Brillouin zone for cells at three different locations: *x* = 19 *mm* (the 1^st^ cell with length Λ_*1*_ = 5.65 *mm*), *x* = 324 *mm* (the 50^th^ cell with length Λ_*50*_ = 6.79 *mm*), and *x* = 693 *mm* (the 100^th^ cell with length Λ_*100*_ = 7.95 *mm*). Within the frequency range in Fig. 2a, there are two types of stopbands: one stopband at point A on the limit of the first Brillouin zone, also known as Bragg stopband, and the other stopband at point B inside the first Brillouin zone. The Bragg stopband at point A is induced by the Bragg reflection of A0 mode. This stopband breaks the dispersion curve of A0 mode, which means the A0 mode is not allowed to propagate through it. In contrast, the S0 mode can still propagate through the Bragg stopband of A0 mode for the reason that its dispersion curve keeps intact. The other stopband at point B is generated by the coupling between S0 and A0 modes and referred to as ‘S0-A0 stopband’. This one is a full stopband that breaks the dispersion curves of both A0 and S0 modes, which means both A0 and S0 modes are not allowed to propagate in the stopband. Details of the ‘S0-A0 stopband’ can be found in the supplementary document.

Since the S0-A0 stopband is a full stopband that stops both the wave modes, it is used for designing the chirped phononic crystal plate. Figure 2b plots the frequency variation of S0-A0 stopband with respect to *x* location in the chirped phononic crystal plate. With the increase of location *x*, the band width gradually becomes smaller, and the stopband gradually shifts to lower frequencies. Hence, if guided waves that carry frequencies in the range 130~180 kHz enter the phononic crystal plate from the 1^st^ cell and propagate in the +*x* direction, different frequency components (for example *f*_1_ and *f*_2_ plotted in Fig. 2b) will arrive at different locations (*x*_1_ and *x*_2_) on the lower boundaries of the local stopband. At the locations on the lower boundaries, waves will stop forward propagating, turn around, and start propagating backward.

**Figure 3:**
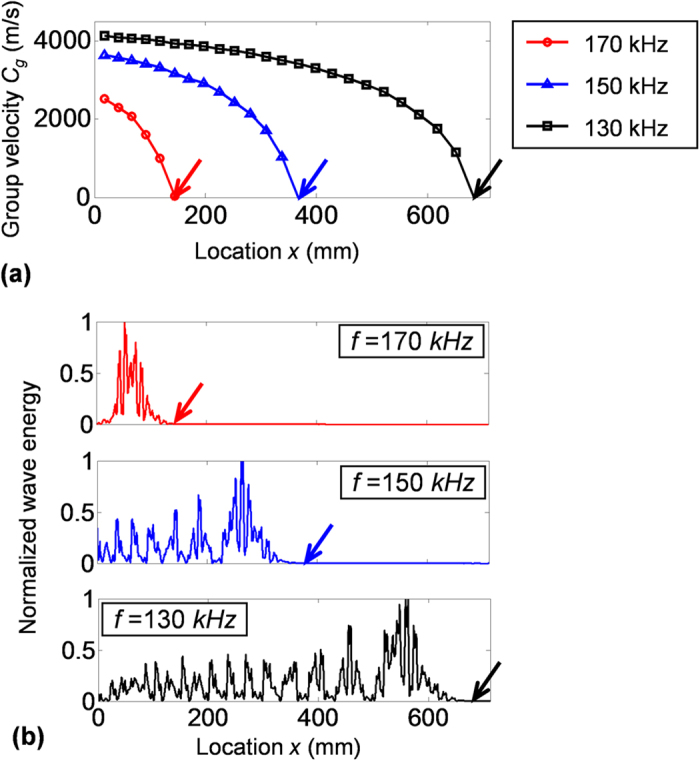
(**a**) Group velocities versus location *x* in the chirped phononic crystal plate at frequencies of 130, 150 and 170 kHz. Group velocities gradually slow down along the +*x* direction, and eventually reach zero (marked with colored arrows). (**b**) Spatial wave energy distributions along the top surface of the plate at frequencies of 130, 150 and 170 kHz. The simulation results show waves of different frequencies stop propagating forward at different locations (marked with colored arrows), which agree well with locations of zero group velocities (marked with colored arrows in figure **a**).

Group velocity *C*_*g*_ is derived as well by calculating the slope (*df*/*dk*) of frequency-wavenumber dispersion curve. Figure 3a plots group velocities of S0 mode with respect to location *x* in the chirped phononic crystal plate at three selected frequencies 130, 150 and 170 kHz. As shown in Fig. 3a, the group velocities gradually decrease with the increase of location *x*, and eventually reach zero at certain locations for different frequencies. Note that the locations of zero group velocities are on the lower boundaries of their corresponding stopbands. At the lower boundaries, the dispersion curves have zero slope (i.e., *df*/*dk* = 0), and thus the corresponding group velocities *C*_*g*_ are zero, i.e., *C*_*g*_ = 2*π* · *df*/*dk* = 0^16^. The group velocity curves in Fig. 3a indicate that when waves of different frequencies propagate forward along the plate, the waves can gradually slow down and stop propagating forward at different locations of zero group velocities. Moreover, since waves propagate slower in the low velocity region, it is expected that waves are more concentrated in space and the corresponding spatial wave energy is therefore considered being enhanced in the low velocity region.

### Numerical simulations

To evaluate the ultrasonic guided waves in the chirped phononic plate given in Fig. 1a, finite element simulations are performed by using the ANSYS Multiphysics 11.0. A wafer type piezoelectric actuator (thickness 0.3 mm and width 5 mm) is bonded on the top surface at the left end of the plate to generate ultrasonic guided waves. The excitation is a chirp signal from 130 kHz to 180 kHz.

The spatial wave energy distribution (determined by *ρω*^2^*u*^2^ where *ρ* is the density, *ω* is the angular frequency, *u* is the displacement^17^) along the top surface of the plate at frequencies of 130, 150 and 170 kHz are calculated and plotted in Fig. 3b. Compared to theoretical group velocities in Fig. 3a, it can be found that the simulation results agree well with the predictions from group velocities. First, the locations (marked with colored arrows) where waves stop propagating forward in simulations agree well with the locations of zero group velocities. Second, as expected, the simulation results show high wave energy concentrated in the low velocity region, which has also been pointed out from the analysis of spatially variant group velocity. Hence, the region with high wave energy can be estimated graphically using the derived group velocity curves.

**Figure 4:**
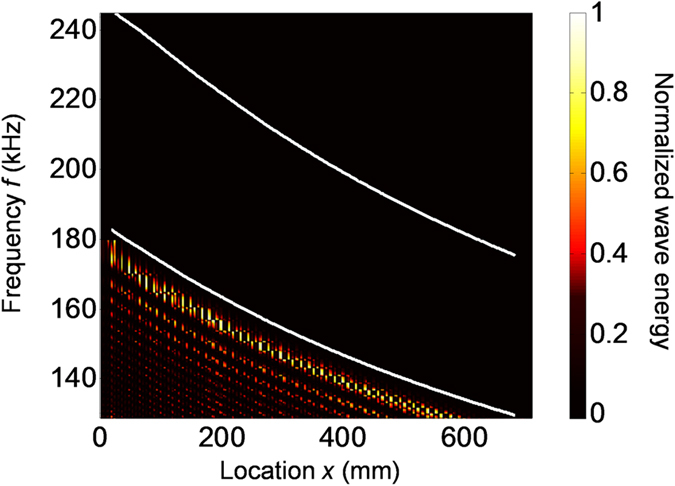
The wave energy distribution with respect to the frequency *f* and location *x*. This figure clearly shows the rainbow trapping of ultrasonic guided waves. Waves at different frequencies stop propagating forward and the corresponding wave energy is concentrated at different locations near the lower boundary of the local stopband. The two white curves represent boundaries of the local stopband obtained from Fig. 2b.

At all excitation frequencies from 130 kHz to 180 kHz, wave energy distributions along the top surface of the plate are calculated. Figure 4 plots a frequency-space representation (or image) of the calculated wave energy with respect to the excitation frequency *f* and location *x*. The image in Fig. 4 shows that waves at different frequencies stop propagating forward and the corresponding wave energy is concentrated at different locations near the lower boundary of the local stopband. Moreover, with the increase of excitation frequency, the wave propagation distance gradually becomes shorter and the location with concentrated energy gradually shifts to the left, which manifests as rainbow guided waves.

### Experimental test

**Figure 5:**
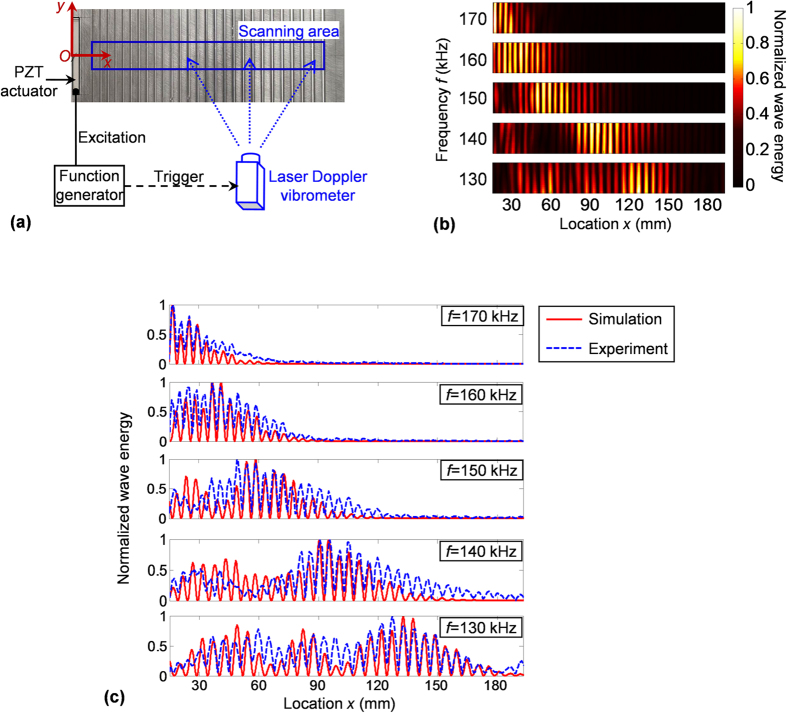
Experimental demonstration. (**a**) Schematic of the experimental setup. A strip PZT actuator is bonded on the left side of the specimen to generate ultrasonic waves. A scanning laser Doppler vibrometer is used to acquire the wavefield in the scanning area. (**b)** Normalized wave energy distributions in the scanning area, at five different frequencies 130, 140, 150, 160 and 170 kHz. Figure (**b**) shows that waves of different frequencies stop propagating forward at different locations. (**c**) Comparisons between experimental and numerical results (normalized energy with respect to location *x*) at five different frequencies 130, 140, 150, 160 and 170 kHz.

A proof-of-concept experiment has been done on a small-scale specimen made of a 4 mm thick aluminum plate. Figure 5a plots the experimental setup. The specimen has 27 milled grooves with width of 2.95 mm and depth of 1.61 mm. The spacing between two consecutive grooves gradually increases from 5.65 mm to 7.95 mm with the same increment 0.092 mm. A strip PZT (lead zirconate titanate) actuator with dimensions of 60 mm × 5 mm × 0.3 mm is bonded on the left side of the specimen to generate ultrasonic guided waves. A scanning laser Doppler vibrometer (model: Polytec PSV-400-M2) is used to acquire the particle velocity *v* in the direction of the laser beam^34^. By measuring velocities at all the predefined points in the scanning area, we can acquire a velocity wavefield in the entire scanning area. From the velocity wavefield, the normalized wave energy can be derived. Figure 5b plots the normalized wave energy acquired at different frequencies of 130, 140, 150, 160 and 170 kHz. The experimental results show that waves of different frequencies stop propagating forward with energy concentrated at different locations. In Fig. 5b, the non-uniform energy distribution along *y* direction is induced by noises and experimental errors that relate to the non-uniform laser reflectivity over the test specimen.

Simulation results for the experimental specimen are provided as well. Figure 5c compares the experimental and numerical results (normalized energy with respect to location *x*) at five selected frequencies 130, 140, 150, 160 and 170 kHz. Overall the experimental and numerical results agree well with each other at all the frequencies, except for some minor differences that might be induced by the experimental errors and noises. The locations where waves stop propagating forward are nearly the same, and the regions with intensive energy agree well with each other.

## Discussion

This study presents rainbow trapping of ultrasonic guided waves in chirped phononic crystal plates with spatially variant dispersion, group velocity, and stopband. Through simulation and experiment, we demonstrated rainbow trapping of guided waves in the frequency range 130~180 kHz. The demonstrated rainbow trapping is related to the progressively slowing group velocity in space, and the spatially variant dispersion characteristics.

Compared to the classic Bragg reflection for a single mode in its Bragg stopband, the rainbow trapping achieves not only the full stopbands of all the modes that are present but more importantly the progressively slowing group velocities in space. As guided waves propagate along the chirped phononic crystal plate, waves gradually slow down due to the progressively slowing group velocity. The energy of guided waves is then concentrated at the low velocity region near the lower boundary of the stopband. Moreover, due to the spatially variant dispersion characteristics, the wave energy of different frequencies can be concentrated at different locations, which manifests as rainbow guided waves. With group velocities curves the energy trapping locations can be estimated graphically. Theoretical formulation for predicting trapping locations is currently being investigated and will be reported in the future.

With the achieved results and experimental demonstration presented in this study, the ultrasonic field will be enlightened for potential interesting work related to ultrasonic guided waves through the ultrasonic rainbow trapping. In guided wave based nondestructive evaluation, the spatially concentrated wave energy will provide enhanced wave-structure interaction effects that are key for extracting defect related wave features. The spatial separation of different frequencies will allow for delivering guided waves of specific frequencies for targeted evaluation. Beyond nondestructive evaluation applications, the rainbow trapping also provides novel ways to accumulate energy for energy harvesting, and manipulate waves in acoustofluidic devices for controlling microparticles and cells, to name a couple here.

## Methods

### Numerical simulations

The 1D chirped phononic crystal plate is made of a 6061-T6 aluminum plate (thickness 4 mm, Young’s modulus 68.9 GPa, Poisson’s ratio 0.33, and density 2700 kg/m^3^). As shown in Fig. 4, along the *x* direction of the plate, there are 101 grooves of the same width *w* = 2.95 mm and depth *d* = 1.61 mm. The cell length *p* gradually increases from 5.65 mm to 7.95 mm with the increment *∆* = 0.023 mm. A piezoelectric actuator (thickness 0.3 mm and width 5 mm) is bonded on the top surface at the left end of the plate to generate ultrasonic waves. Material properties of the piezoelectric actuator can be found in the reference^35^.

A finite element model of the chirped phononic crystal plate is built in the commercial software ANSYS Multiphysics 11.0. The SOLID45 elements are used to build the plate. The coupled field element SOLID5 is selected to construct the piezoelectric actuator. At different excitation frequencies (from 130 kHz to 180 kHz with a step of 1 kHz) of the piezoelectric actuator, the displacement field of guided waves in the model is simulated using ANSYS. Then the spatial wave energy distribution is calculated by *ρω*^2^*u*^2^, where *ρ* is the density, *ω* is the angular frequency, *u* is the displacement^17^.

The dispersion curves (Fig. 2) are calculated by using the finite element method in the commercial software COMSOL Multiphysics 4.4. In finite element method, the meshing sizes are smaller than 1/10 of the smallest wavelength, in order to accurately capture characteristics of waves in the 1D chirped phononic crystal plate.

### Experimental test

The test specimen is made of a 4 mm thick 6061-T6 aluminum plate. On the plate, there are 27 grooves created by a milling machine, which have the same width 2.95 mm and depth 1.61 mm. The spacing between two consecutive grooves gradually increases from 2.7 mm to 5 mm with the same increment 0.092 mm, along the *x* direction of the specimen. A schematic of the experimental setup is given in Fig. 5a. A strip PZT actuator with dimensions of 60 mm × 5 mm × 0.3 mm (manufactured by Steiner & Martins, Inc.) is bonded on the left side of the specimen to generate ultrasonic waves. The PZT excitation signal is generated by an arbitrary waveform generator (Hewlett Packard 33120A). A scanning laser Doppler vibrometer (model: Polytec PSV-400-M2) is used to acquire the particle velocity *v* in the direction of laser beam based on Doppler Effect^36^,^37^. The laser vibrometer directly measures temporal waves in time-domain, and then the time-signal is transformed to frequency domain by using Fourier transform. Hence, the laser vibrometer can output a scan result as magnitude distribution in the scan area at the selected frequency. In the test, experimental errors can be caused by noise and non-uniform laser reflectivity of the test specimen. Note that in the specimen preparation we have sanded and cleaned the specimen in order to reduce the errors caused by non-uniform reflectivity.

## Additional Information

**How to cite this article**: Tian, Z. and Yu, L. Rainbow trapping of ultrasonic guided waves in chirped phononic crystal plates. *Sci. Rep.* **7**, 40004; doi: 10.1038/srep40004 (2017).

**Publisher's note:** Springer Nature remains neutral with regard to jurisdictional claims in published maps and institutional affiliations.

## Supplementary Material

Supplementary Information
