# Supplementary material for: Rainbow trapping of ultrasonic guided waves in chirped phononic crystal plates
Source: Sci Rep. 2017 Jan 5;7:40004. doi: 10.1038/srep40004 (PMC5213308; doi:10.1038/srep40004)
Supplement: Supplementary Information [file srep40004-s1.pdf]

Rainbow trapping of ultrasonic guided waves in chirped phononic crystal plates

Zhenhua Tian[*], Lingyu Yu

Department of Mechanical Engineering, University of South Carolina, Columbia, SC, 29208, USA
[*] Corresponding author: tianz@email.sc.edu

Supplementary information

Dispersion curves in uniform phononic crystal plates

In free smooth plates, Lamb waves (plate guided waves) propagate in forms of anti-symmetric and symmetric modes [1]. When a smooth plate has periodical grooves, Lamb waves in the plate will be affected by the periodical grooves [2]. For example, stopbands will present in the grooved plate. This section presents derivations of dispersions curves for smooth and grooved plates, respectively. The relation between waves in smooth and grooved plates is investigated. Moreover, the analytical approximation of stopband frequency for the grooved plate is derived.

In a smooth plate, Lamb waves have anti-symmetric and symmetric modes. Their dispersion curves can be obtained by solving the Rayleigh-Lamb equations[1]:

$$\frac{\tan(qt/2)}{\tan(pt/2)} = -\frac{4k^2qp}{(k^2-q^2)^2} \text{ for symmetric modes} \tag{S1}$$

$$\frac{\tan(qt/2)}{\tan(pt/2)} = -\frac{(k^2-q^2)^2}{4k^2qp} \text{ for anti-symmetric modes} \tag{S2}$$

where

$$p^2 = \frac{\omega^2}{c_L^2} - k^2,\ q^2 = \frac{\omega^2}{c_S^2} - k^2,\ c_L = \sqrt{\frac{2\mu(1-\nu)}{\rho(1-2\nu)}} \text{ and } c_S = \sqrt{\frac{\mu}{\rho}}.$$

k and ω are wavenumber and circular frequency. c_L and c_S are velocities of longitudinal and transverse waves. ρ, μ and ν are density, shear modulus and Poisson's ratio of the material, respectively. t is plate thickness. By solving the Rayleigh-Lamb equations, the dispersion relation between wavenumber k and frequency $f=\omega/2\pi$ can be obtained. Figure S1a plots theoretical

dispersion curves for Lamb waves in a 2.39 mm thick aluminum plate. In the frequency range 0~300 kHz, two wave modes are exhibited: anti-symmetric A0 and symmetric S0 modes. Figure S1b plots folded dispersion curves obtained by folding the curves in Figure S1a at wavenumbers k=0 and k=π/Λ (Λ = 7.95 *mm*). The folded dispersion curves will be used later and compared to dispersion curves for a grooved plate.

For a plate waveguide with infinite periodical grooves along the x direction (as shown in Figure S2), the waveguide can be considered as a 1D uniform phononic crystal plate. To analytical derive the dispersion curves for periodical grooved plates, reflective array method and coupled-mode method have been used in literatures [3,4]. However, these analytical methods only provide approximations of dispersion curves. In addition, when there are multiple wave modes that interact with each other, the analytical methods have limitations to derive their dispersion curves. Therefore, most studies adopt numerical methods to solve the dispersion curves, especially for periodical structures with complex-shape cells [5-8]. Moreover, dispersion curves solved by numerical methods are more precise than analytical approximations for complex-shape cells. In this study, dispersion curves are derived by solving a modal analysis problem on a unit cell with Bloch-Floquet condition written as:

$$F(x+\Lambda) = \mathrm{e}^{j\Lambda k} \cdot F(x) \tag{S3}$$

where F is any space function (stress, displacement, etc.), k is wavenumber, and Λ is cell length. Bloch-Floquet boundary conditions are defined on the left (x=0) and right (x=Λ) boundaries of a unit cell, as shown in Figure S2. By solving the modal analysis problem on a unit cell, we can find the frequency f for a given wavenumber k and then obtain the dispersion relation $f(k)$. In this study, the modal analysis problem is solved in the commercial finite element software COMSOL Multiphysics 4.4. The solved dispersion relation $f(k)$ is 2π/Λ periodic with respect to the wavenumber k, i.e., $f(k)= f(k+2\pi/\Lambda)$. This relation implies that the dispersion study can be limited to wavenumbers in the first Brillouin zone.

Figure S3a plots dispersion curves in the first Brillouin zone for a grooved aluminum plate with parameters t=2.39 mm, d=0.1 mm, w=2.95 mm, Λ=7.95 mm, and depth/total thickness ratio d/h = 0.04. The dispersion curves exhibit two modes. We follow the notation in the reference[6] and denote the two modes as pseudo-Lamb modes A0^Λ, and S0^Λ. The dispersion curves (in Figure S3a) for the grooved plate and the folded dispersion curves (in Figure S1b) for the smooth plate are plotted together in Figure S3b for comparison purpose. It can be seen that dispersion curves for smooth and

grooved plates are nearly the same. Hence, the theoretical dispersion curves for Lamb waves in smooth plates can be used as approximations of dispersion curves for grooved plates, especially when the depth/total thickness ratio is very small. This statement can also be confirmed from other studies [2,9].

The dispersion curves for the grooved plate in Figure S3b clearly exhibit two types of stopbands: one stopband at point A on the limit of the first Brillouin zone, also known as Bragg stopband, and the other stopband at point B inside the first Brillouin zone. The stopband at point A is related to the opening of a gap at the folding of A0 dispersion curve, which is induced by Bragg reflection and the coupling between forward and backward propagating A0 modes[2]. This stopband breaks the dispersion curve of A0 mode, which means the A0 mode is not allowed to propagate through it. In contrast, the S0 mode can propagate through the Bragg stopband of A0 mode, since its dispersion curve keeps intact. Using the Lamb wave approximation, the stopband frequency at point A can be approximated using,

$$f_A = f_{A0}(k_A = \pi/\Lambda) \tag{S4}$$

where f_{A0} is the dispersion relation of A0 mode. As shown in the zoomed-in plot at point A, the approximation at the folding point of A0 dispersion curve is within the Bragg stopband.

The stopband at point B is related to the opening of a gap at the crossing of A0 and S0 dispersion curves[2] and referred to as “S0-A0 stopband”. From the dispersion curves, it can be seen that this stopband is induced by the coupling between forward propagating S0 and backward propagating A0 modes. Moreover, it is a full stopband that breaks dispersion curves of both A0 and S0 modes, which means both A0 and S0 modes are not allowed to propagate in the stopband. Using the Lamb wave approximation, the S0-A0 stopband frequency at point B can be approximated using,

$$f_B = f_{A0}(2\pi/\Lambda - k_B) = f_{S0}(k_B) \tag{S5}$$

where f_{S0} is the dispersion relation of S0 mode. As shown in the zoomed-in plot at point B, the approximation at the intersection of A0 and S0 dispersion curves is within the S0-A0 stopband.

The effects of depth/total thickness ratio (d/h) on stopband frequencies are investigated parametrically. Figure S4a plots the variations of Bragg and S0-A0 stopbands with respect to d/h in the range 0.04~0.4. With the increase of d/h, the widths of Bragg and S0-A0 stopbands gradually expand, and the lower boundaries of both stopbands gradually drop. The two approximations are always within their corresponding stopbands at different values of d/h.

The effects of spacing/cell length ratio (s/Λ) on stopband frequencies are also investigated parametrically. Figure S4b plots the variations of Bragg and S0-A0 stopbands with respect to s/Λ ratio in the range 0.47~0.9. With the increase of s/Λ, the widths of Bragg and S0-A0 stopbands gradually shrink, and the frequencies of both stopbands gradually drop. The two approximations also gradually drop and nearly follow the trends of their corresponding stopbands.

Stopbands in a 1D chirped phononic crystal plate

A 1D chirped phononic crystal plate is considered, which is made of a 4 mm thick aluminum plate with an array of 101 grooves. The cross-section of the plate is plotted in Figure S5a, and three consecutive cells n-1, n and n+1 are plotted in Figure S5b. All grooves have the same width w= 2.95 mm and depth d= 1.61 mm. Along the x direction of the plate, the cell length Λ_n linearly increases from 5.65 mm to 7.95 mm with the same increment Δ = 0.023 mm. The length and location of the nth cell can be expressed as

$$\Lambda_n = \Lambda_1 + (n-1)\Delta\,,\ \ x_n = x_0 + \frac{(n-1)^2\Delta + (2n-1)\Lambda_1}{2} \tag{S6}$$

where Λ_n and x_n are the length and center location of the nth cell. x_0 is the location of the first cell's left boundary. Since the increment Δ between two consecutive cells is very small (0.023 mm), we could consider the nth cell with length Λ_n in chirped phononic crystals as a cell with the same length Λ_n in uniform phononic crystals. Therefore, we could use dispersion characteristics of the uniform phononic crystals to approximate dispersion characteristics at the nth cell in the chirped phononic crystals.

Since the cell length Λ_n linearly increases along the x direction in the chirped phononic crystal plate, cells at different locations will have different frequency-wavenumber dispersion curves and stopbands. Figure S6 plots the variations of Bragg and S0-A0 stopbands with respect to location x in the plate. With the increase of location x, both stopbands gradually shift to lower frequencies. Using Eqs. (S4), (S5) and (S6), approximations of Bragg and S0-A0 stopbands are derived and then plotted in Figure S6. The two approximations gradually drop with the increase of location x and nearly follow the trends of their corresponding stopbands.

Transient wave propagation in a 1D chirped phononic crystal plate

To investigate the transient wave propagation in a 1D chirped phononic crystal plate, transient guided waves in the plate are simulated by using finite element method. Figure S7 plots the simulation setup. A wafer type piezoelectric actuator (thickness 0.3 mm and width 5 mm) is bonded on the top surface at the left end of the plate to generate ultrasonic guided waves. The material properties of the piezoelectric actuator can be found in the reference[10]. The excitation is a signal of 40-count tone bursts at 150 kHz. This frequency is selected for illustration purpose, and transient waves at other frequencies could also be simulated using the same method.

Figure S8b plots the simulation results (spatial distributions of displacement $u_y(x)$ along the bottom of the plate) at different time from 60 μs to 440 μs with a step of 20 μs (3 circles at 150 kHz), which directly shows the propagation of guided waves in the plate. The theoretical group velocity of $S0^{\Lambda}$ mode with respect to location x at the excitation frequency 150 kHz is plotted in Figure S8a for comparison purpose.

From 60 μs to 180 μs, the generated guided waves propagate forward. After 180 μs, waves enter the relatively low velocity region 200~360 mm, gradually become slower, and stop propagating forward at 360 mm, the location of zero group velocity. From 180 μs to 280 μs, it can be seen that the wave amplitude in the low velocity region gradually increases due to the trapping and accumulation of low velocity waves. In the time range 280 ~ 380 μs, the waves are localized in the low velocity region manifesting as ‘trapped waves’. Moreover, the amplitude of trapped waves remains at a high level, which is nearly two times of the propagating waves in the region 0~200 mm and in the time range 80~180 μs. After 380 μs, the waves trapped in the low velocity region are gradually released from the low velocity region and then propagating backward.

Figure S9 plots a zoomed-in view of simulation results in the low velocity region 200~360 mm and in the time range 280~380 μs. From the zoomed-in plot, it can be seen that waves at different time (with a step of 3 periods $3T$) have nearly the same amplitude and phase information. These observations are attributed not only to the extreme low group velocity, but also to the coupling between the forward and backward waves (reflected by the stopband). The forward incident waves and backward reflected waves interfere with each other and form standing waves, i.e., the waves plotted in Figure S9. The generation of standing waves further strengthens the wave amplitude.

From the simulation and analysis results, it can be found that the transient wave propagation in the 1D chirped phononic crystal plate includes four stages. (1) Incident waves generated from the

actuator propagate forward. (2) Incident waves enter the low velocity region, slowing down and then trapped. The wave amplitude in the low velocity region gradually increases due to the trapping and accumulation of low velocity waves. (3) Waves are continuously trapped in the low velocity region. In addition, forward incident waves and backward reflected waves (reflected by the stopband) interfere with each other and form standing waves that further strengthen the wave amplitude. (4) The trapped waves are gradually released and propagate backward.

References

1 Rose, J. L. *Ultrasonic Waves in Solid Media*. (Cambridge University Press, 1999).

2 Bavencoffe, M., Hladky-Hennion, A. C., Morvan, B.Izbicki, J. L. Attenuation of Lamb Waves in the Vicinity of a Forbidden Band in a Phononic Crystal. *Ieee Transactions on Ultrasonics Ferroelectrics and Frequency Control* **56**, 1960-1967 (2009).

3 Morgan, D. *Surface Acoustic Wave Filters with Applications to Electronic Communications and Signal Processing*. (Academic Press, 2007).

4 Plessky, V.Koskela, J. Coupling-of-modes Analysis of SAW Devices. *International Journal of High Speed Electronics and Systems* **10**, 867-947 (2000).

5 Langlet, P., Hladkyhennion, A. C.Decarpigny, J. N. Analysis of the Propagation of Plane Acoustic-Waves in Passive Periodic Materials Using the Finite-Element Method. *Journal of the Acoustical Society of America* **98**, 2792-2800 (1995).

6 Bavencoffe, M., Morvan, B., Hladky-Hennion, A. C.Izbicki, J. L. Experimental and numerical study of evanescent waves in the mini stopband of a 1D phononic crystal. *Ultrasonics* **53**, 313-319 (2013).

7 Predoi, M. V., Castaings, M., Hosten, B.Bacon, C. Wave propagation along transversely periodic structures. *Journal of the Acoustical Society of America* **121**, 1935-1944 (2007).

8 Charles, C., Bonello, B.Ganot, F. Propagation of guided elastic waves in 2D phononic crystals. *Ultrasonics* **44**, E1209-E1213 (2006).

9 Morvan, B., Hladky-Hennion, A. C., Leduc, D.Izbicki, J. L. Ultrasonic guided waves on a periodical grating: Coupled modes in the first Brillouin zone. *Journal of Applied Physics* **101**, 114906 (2007).

10 Song, F., Huang, G. L.Hudson, K. Guided wave propagation in honeycomb sandwich structures using a piezoelectric actuator/sensor system. *Smart Materials & Structures* **18**, 125007 (2009).

Figures

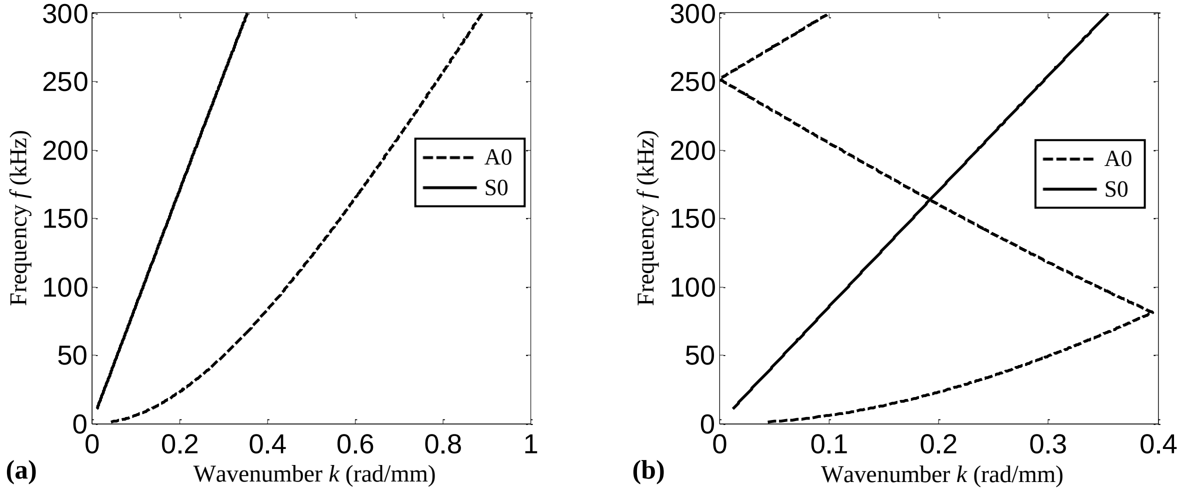

Figure S1. Dispersion curves for Lamb waves in a 2.39 mm thick aluminum plate: (a) dispersion curves solved from Rayleigh-Lamb equations; (b) dispersion curves folded at k=0 and k=π/Λ (Λ = 7.95 *mm*).

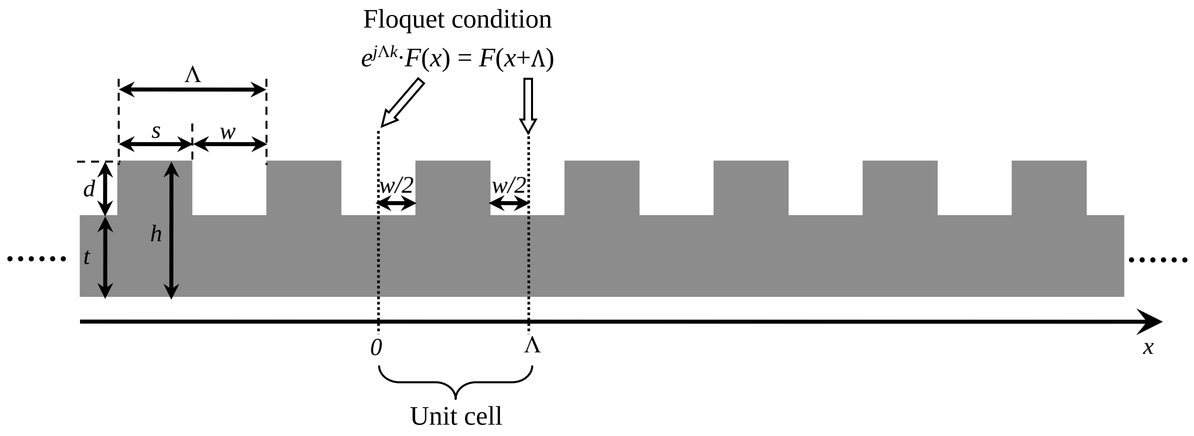

Figure S2. Illustration of a 1D uniform phononic crystal plate.

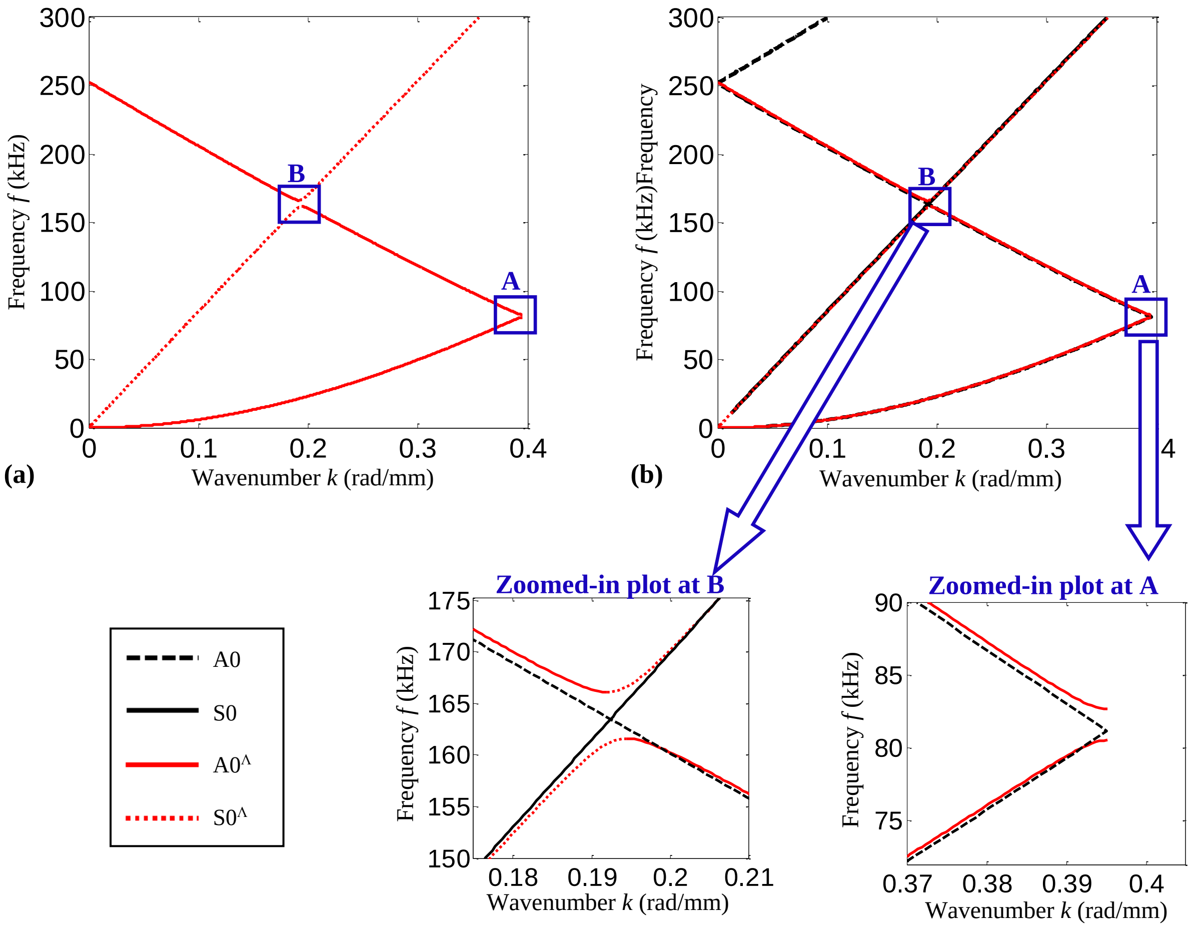

Figure S3. Dispersion curves in the first Brillouin zone for a grooved aluminum plate with parameters t=2.39 mm, d=0.1 mm, w=2.95 mm, and Λ=7.95 mm. The depth/thickness ratio of the grooved plate is 0.04. (a) Dispersion curves for the grooved plate exhibit two modes $A0^{\Lambda}$ and $S0^{\Lambda}$. (b) The comparison between dispersion curves for smooth and grooved plates shows that dispersion curves in two plates are nearly the same.

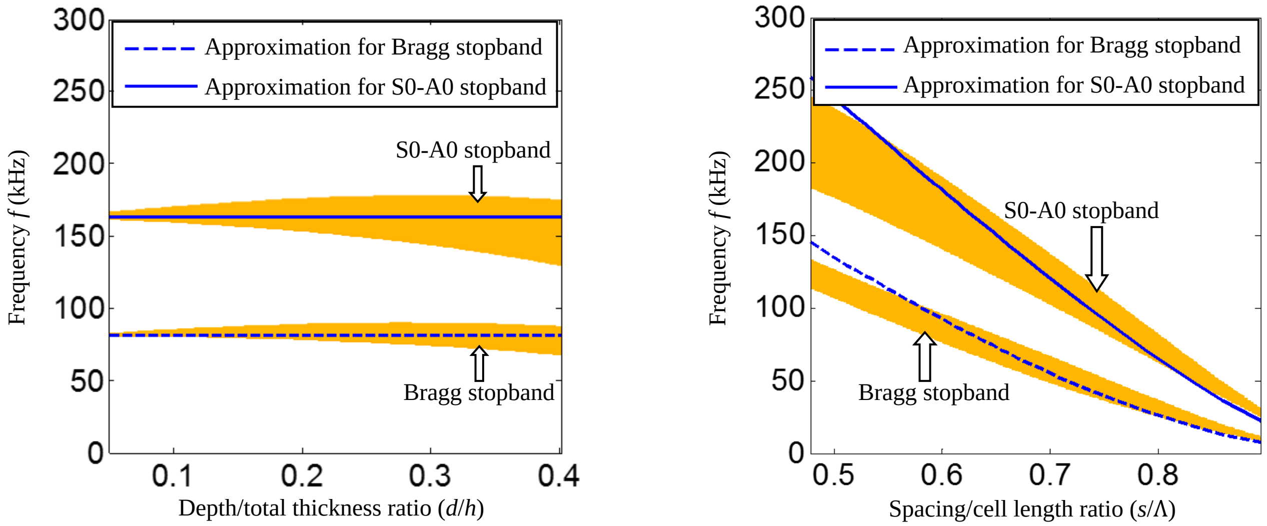

Figure S4. Variations of Bragg and S0-A0 stopbands with respect to (a) depth/total thickness ratio (d/h), and (b) spacing/cell length ratio (s/Λ). The shaded areas represent stopbands derived by using finite element method. The dash and solid lines are Lamb wave approximations for Bragg and S0-A0 stopbands, respectively.

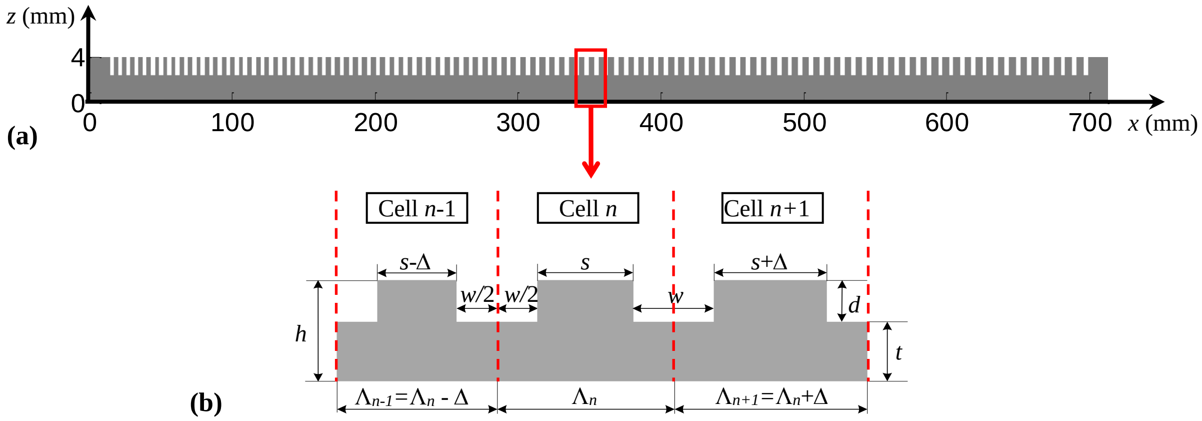

Figure S5. Schematics for a chirped phononic crystal plate. (a) The cross-section of the plate. (b) Three consecutive cells in the plate. All grooves in the plate have the same width *w* and depth *d*. The spacing *s* between two consecutive grooves is linearly increasing with the same increment Δ, along the *x* direction.

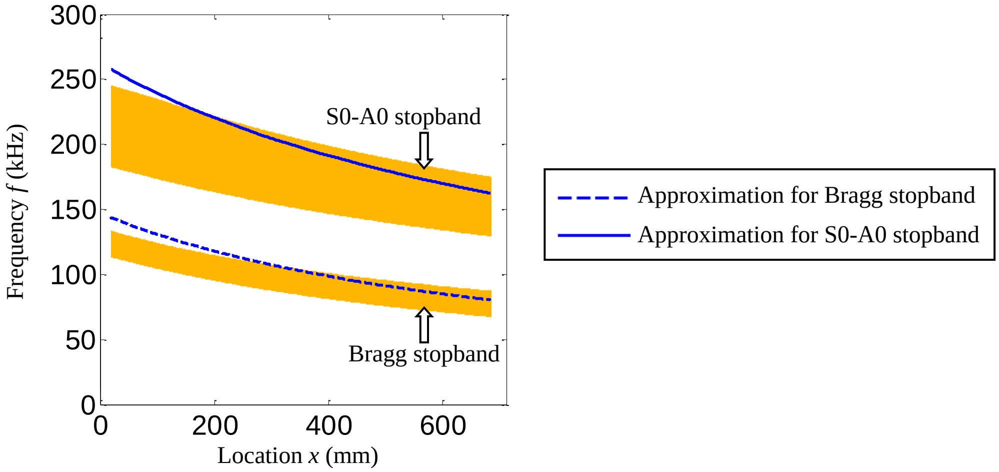

Figure S6. Variations of Bragg and S0-A0 stopbands with respect to location *x* in the 1D chirped phononic crystal plate. The shaded areas represent stopbands derived by using finite element method. The dash and solid lines are Lamb wave approximations for Bragg and S0-A0 stopbands.

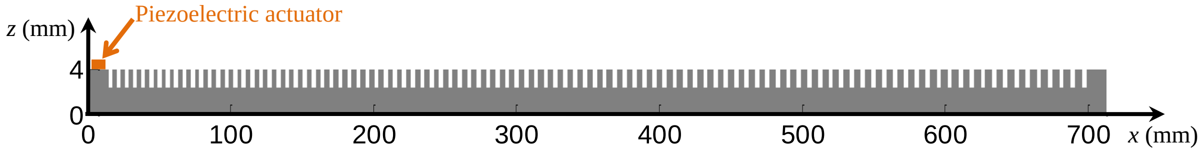

Figure S7. The setup for simulating transient guided waves in a 1D chirped phononic crystal plate. A piezoelectric actuator bonded on the top surface of the left side is used to generate guided waves. The excitation is a signal of 40-count tone bursts at 150 kHz.

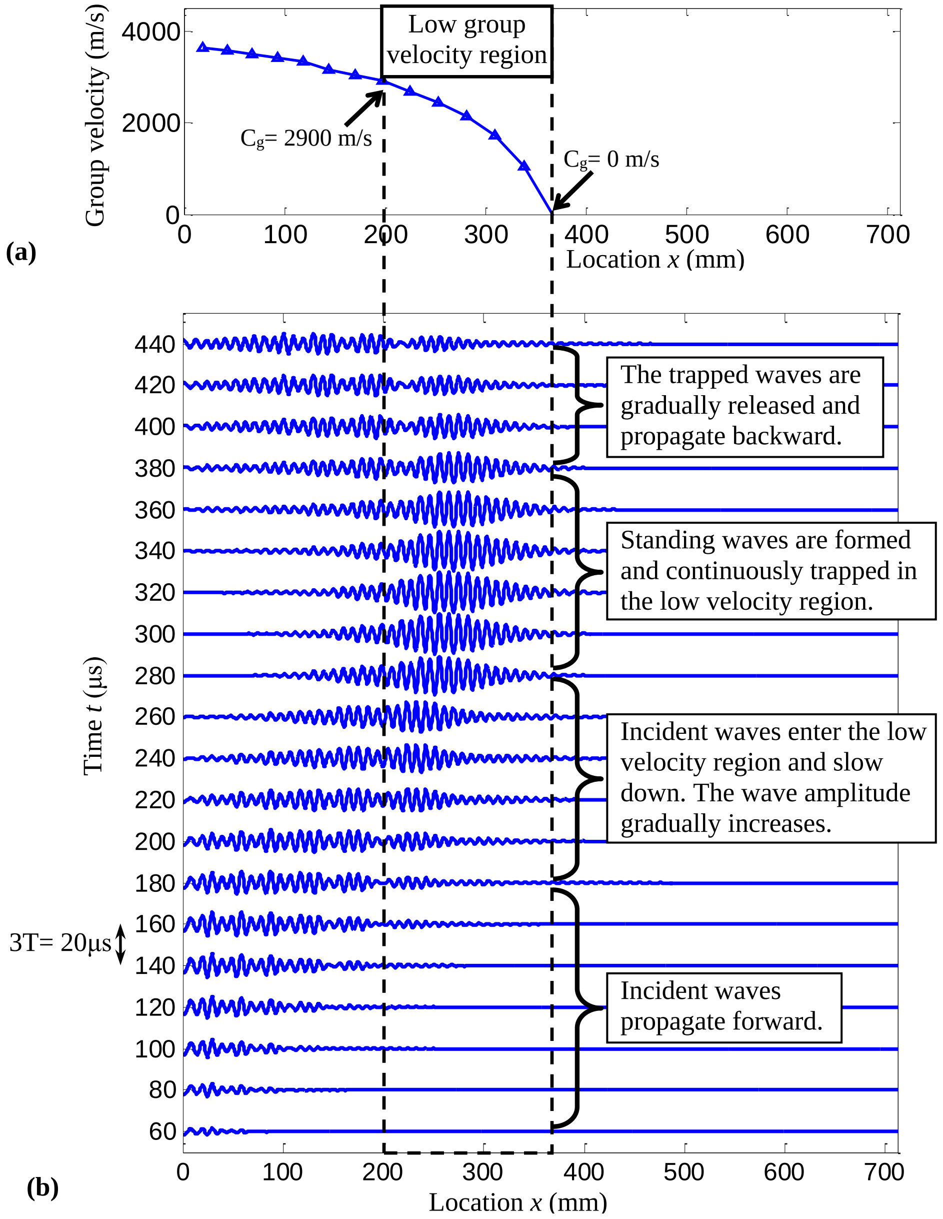

Figure S8. (a) Theoretical group velocity for $S0^{A}$ waves at 150 kHz in the 1D chirped phononic crystals plate. (b) Waterfall plot of simulation results at different time from 60 μs to 440 μs with a step of 20 μs (3 circles at 150 kHz). Each waveform represents the displacement $u_y(x)$ along the plate's bottom at a selected time.

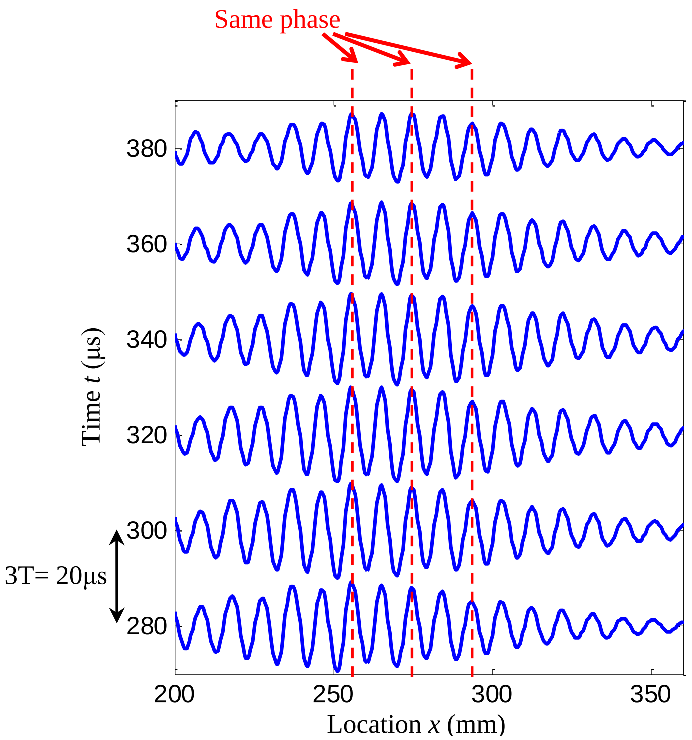

Figure S9. Zoomed-in plot of simulation results in the low velocity region 200~360 mm and in the time range 280~380 μs. The plot shows wave amplitude distributions at different time are nearly the same. In addition, these waveforms have nearly the same phase information. These evidences mean that standing waves are formed due to the interaction of forward incident and backward reflection waves.
